# Initial clinical validation of a hybrid *in silico*—*in vitro* cardiorespiratory simulator for comprehensive testing of mechanical circulatory support systems

**DOI:** 10.3389/fphys.2022.967449

**Published:** 2022-10-13

**Authors:** Libera Fresiello, Kavitha Muthiah, Kaatje Goetschalckx, Christopher Hayward, Maria Rocchi, Maxime Bezy, Jo P. Pauls, Bart Meyns, Dirk W. Donker, Krzysztof Zieliński

**Affiliations:** ^1^ Cardiovascular and Respiratory Physiology, University of Twente, Enschede, Netherlands; ^2^ Department of Cardiovascular Sciences, Katholieke Universiteit Leuven, Leuven, Belgium; ^3^ Department of Cardiology, St Vincent’s Hospital, Sydney, NSW, Australia; ^4^ Department of Cardiovascular Diseases, University Hospitals Leuven, Leuven, Belgium; ^5^ Victor Chang Cardiac Research Institute, Sydney, NSW, Australia; ^6^ School of Engineering, Griffith University, Southport, QLD, Australia; ^7^ Intensive Care Center, University Medical Center Utrecht, Utrecht, Netherlands; ^8^ Nalecz Institute of Biocybernetics and Biomedical Engineering, Polish Academy of Sciences, Warsaw, Poland

**Keywords:** cardiorespiratory simulator, lumped parameter model, 0D models, *in vitro* simulator, exercise physiology, ventricular assist device, mechanical circulatory support, artificial organs

## Abstract

Simulators are expected to assume a prominent role in the process of design—development and testing of cardiovascular medical devices. For this purpose, simulators should capture the complexity of human cardiorespiratory physiology in a realistic way. High fidelity simulations of pathophysiology do not only allow to test the medical device itself, but also to advance practically relevant monitoring and control features while the device acts under realistic conditions. We propose a physiologically controlled cardiorespiratory simulator developed in a mixed *in silico*-*in vitro* simulation environment. As inherent to this approach, most of the physiological model complexity is implemented *in silico* while the *in vitro* system acts as an interface to connect a medical device. As case scenarios, severe heart failure was modeled, at rest and at exercise and as medical device a left ventricular assist device (LVAD) was connected to the simulator. As initial validation, the simulator output was compared against clinical data from chronic heart failure patients supported by an LVAD, that underwent different levels of exercise tests with concomitant increase in LVAD speed. Simulations were conducted reproducing the same protocol as applied in patients, in terms of exercise intensity and related LVAD speed titration. Results show that the simulator allows to capture the principal parameters of the main adaptative cardiovascular and respiratory processes within the human body occurring from rest to exercise. The simulated functional interaction with the LVAD is comparable to the one clinically observed concerning ventricular unloading, cardiac output, and pump flow. Overall, the proposed simulation system offers a high fidelity *in silico*-*in vitro* representation of the human cardiorespiratory pathophysiology. It can be used as a test bench to comprehensively analyze the performance of physically connected medical devices simulating clinically realistic, critical scenarios, thus aiding in the future the development of physiologically responding, patient-adjustable medical devices. Further validation studies will be conducted to assess the performance of the simulator in other pathophysiological conditions.

## 1 Introduction

The role of simulators in the development, testing and regulatory submission of medical devices is constantly growing and strongly encouraged by competent authorities (Morrison et al., 2018). This is of great importance especially for mechanical circulatory support (MCS) systems including ventricular assist devices, total artificial hearts and the vastly evolving percutaneous temporary circulatory support devices. Both the European commission and the Food and Drug Administration are encouraging the development of simulators able to offer a valid and equivalent alternative to animal models for medical devices testing (Avicenna Roadmap, 2019; U.S. Food and Drug Administration, 2019; VPH Institute, 2021). Large animal studies, so far a prerequisite for novel MCS systems, pose significant ethical issues and imply high costs. Moreover, testing is usually conducted in young healthy animals, that cannot completely reflect the complex nature of heart failure and associated disease as clinically observed in patients (Shin et al., 2021).

Physical simulators such as mock loops can be a viable solution for MCS testing. But in order to reduce animal experiments, they need to meet realistic physiological requirements (Fraser et al., 2018). Over the years, mock loops have evolved progressively to more sophisticated systems, starting from the representation of one side of the circulation only (Pantalos et al., 2004) to more comprehensive and integrated configurations including both the systemic and the pulmonary circulation (Timms et al., 2011). Modern *in vitro* simulators can include both arterial and venous compartments, a full four-chamber heart featuring realistic Frank-Starling relations (Dewi et al., 2020) and autonomic controls, to mimic a more realistic reaction of the human cardiovascular system to various MCSs (Ochsner et al., 2013; Jansen-Park et al., 2016). But all in all, *in vitro* simulators are confined to the cardiovascular system, and only a few include a ventilation model but with no respiratory representation (Vukicevic et al., 2013; Tree et al., 2018).

In parallel to that, *in silico* lumped parameter simulators have evolved over the years at a higher pace, thanks to the advancements in computer technology. Current *in silico* simulators include closed-loop real-time representation of both the cardiovascular and respiratory systems fully integrated with each other (Batzel et al., 2007; Broomé et al., 2013; Albanese et al., 2015; Fresiello et al., 2016). The *in silico* simulators capture great complexity, include a multitude of interacting physiological parameters, control mechanisms and non-linearities that ultimately cumulate into a higher fidelity compared to their *in vitro* alter ego. To capture this physiological complexity could be of great utility for *in vitro* systems, when aiming for a high-fidelity test bench for MCSs testing. It is not merely relevant to assess the hemodynamic performance of the MCS itself, but more so its proper integrability into the human body in critical, life-threatening conditions. This would ultimately allow to optimize MCSs performance and to design more effective monitoring and control strategies in patient-specific settings (Parvinian et al., 2018).

From an engineering perspective, it is challenging to translate the complexity of *in silico* cardiorespiratory simulators into an *in vitro* setup. The implementation of a high-fidelity human cardiorespiratory model in a test bench would imply to deal with an overall high model complexity. To bypass these challenges, the possibility to combine *in silico* and *in vitro* systems together have been explored: the *in silico* model retains the high complex physiological model and the *in vitro* system offers the hardware interface to connect and test a medical device (Zielinski et al., 2016; Fresiello and Zieliński, 2020).

In this paper, we adopt this modeling strategy and present a fully integrated *in silico-in vitro* simulator of the human cardiorespiratory system, exhibiting an unprecedented level of fidelity and physiological complexity. The *in vitro* component consists of a hydraulic interface where MCSs can physically be connected. The *in silico* component represents a comprehensive cardiorespiratory model featuring all principal physiological parameters and control mechanisms. As a relevant and exemplary case scenario chronic, severe heart failure supported by a left ventricular assist device (LVAD) was studied under rest and exercise conditions, to demonstrate the versatile and practically meaningful functionality of the simulator. This work constitutes the first fully controlled closed-loop *in silico*-*in vitro* cardiorespiratory simulator, which allows to assess the performance of a real MCS under clinically realistic, complex pathophysiological conditions as encountered during physical exercise in LVAD patients.

## 2 Materials and methods

### 2.1 General simulator structure

**FIGURE 1:**
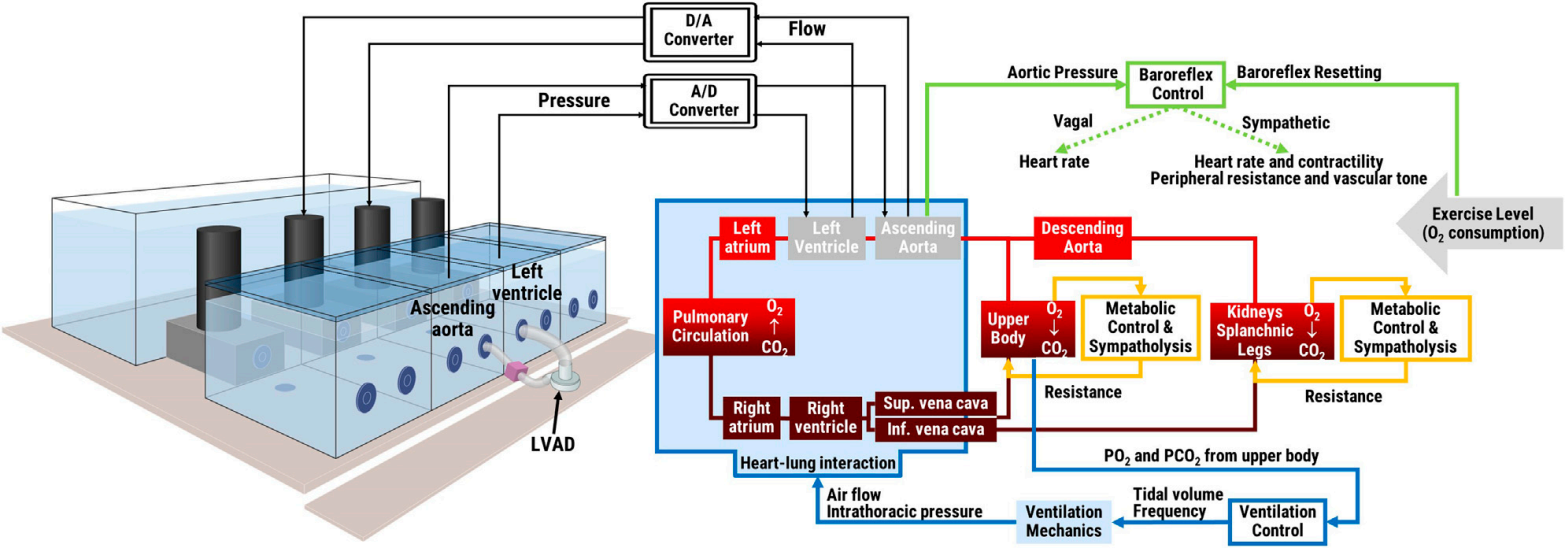
Schematic of the hybrid simulator: *in vitro* interface with an LVAD connected between two hydraulic chambers (left panel) and *in silico* lumped parameters cardiorespiratory model and its main controls (right panel). The two middle hydraulic chambers represent the left ventricle and the aorta and exchange pressure and flow data in real-time with the remainder *in silico* cardiorespiratory model. Physical exercise level is input into the system as oxygen consumption.

The simulator was developed in accordance with an *in silico*—*in vitro* simulation philosophy coined as the hybrid simulation technique (Zielinski et al., 2016; Zieliński et al., 2022): a computational model is combined with a hydraulic interface and pressure and flow data are exchanged in real-time between the two. In our case the computational model is a lumped parameter representation of the cardiovascular and respiratory systems and related controls. It is implemented in LabVIEW 2019 (National Instruments, Austin, TX, United States) by differential equations solved using the Euler method with a time step of 1 ms. The *in silico* model exchanges pressure and flow data in real-time with the *in vitro* interface. The latter includes four active hydraulic chambers that can mimic pressures/flows in up to four sites within the cardiovascular system. The specific configuration of the hydraulic chambers can be adjusted to the requirements of the clinical scenario and particular medical device to be tested (Fresiello and Zieliński, 2020). An overview of the hybrid simulator structure is reported in Figure 1, more details about its components are reported in the next paragraphs.

### 2.2 *In silico* cardiorespiratory model

#### 2.2.1 Cardiovascular model

**FIGURE 2:**
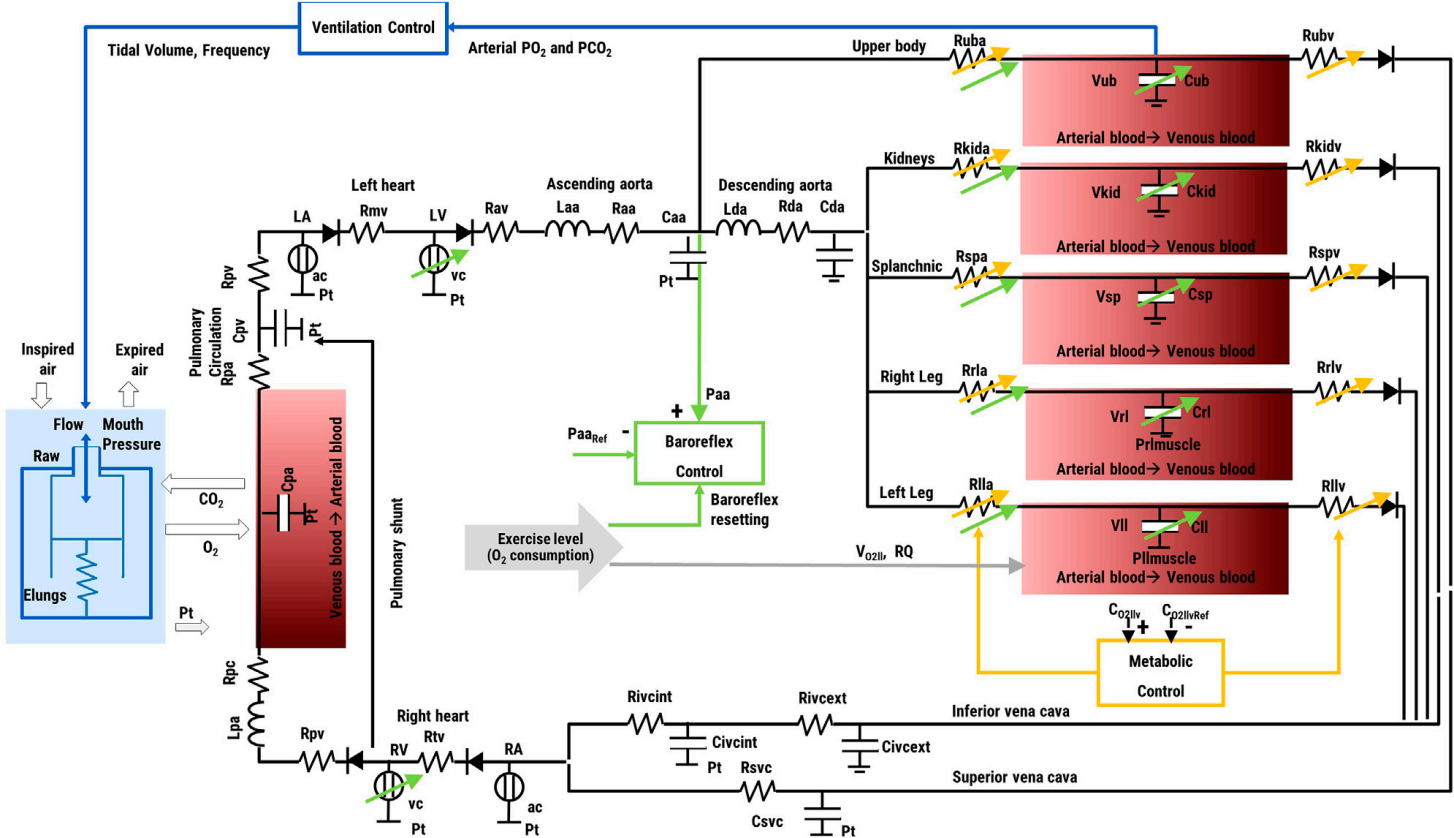
Schematic of the *in silico* simulator. Left and right atria (*LA*, *RA*) and ventricles (*LV*, *RV*) contraction is regulated by activation functions (*ac*, *vc*). Valves are represented by a diode and a resistance (*Rmv*, *Rav*, *Rtv*, *Rpv*). The i_th_ vascular compartment is represented by resistance (*Ri*), inertance (*Li*), and compliance (*Ci*). Suffix refer to: ascending and descending aorta (*aa*, *da*), upper body (*ub*), kidneys (*kid*), splanchnic circulation (*sp*), left and right legs (*ll*, *rl*), inferior and superior vena cava (*ivc*, *svc*), arterial, and pulmonary circulation (*pa*, *pv*). The baroreflex control uses the difference ascending aortic pressure (*Paa*)—reference pressure (*Paa*_*Ref*_) as input, and controls heart rate, heart contractility, arterial resistances and venous tone. The metabolic control acts locally in the upper body, kidneys, splanchnic circulation and legs. In the figure it is represented only for the left leg for brevity. It takes the difference O_2_ concentration (*C*_*O2llv*_)—reference concentration (*C*_*O2llvRef*_) as input, and controls *R*_*lla*_ and *R*_*llv*_ as output. The ventilation control takes the arterial O_2_ and CO_2_ partial pressure in the upper body as input and controls the tidal volume and ventilation frequency as output. The ventilation model includes a resistance (*Raw*) and a compliance (*Elungs*) and provides the intrathoracic pressure (*Pt*) as output, sent to the vessels of the circulation model within the thorax. The model of gas exchange in the lungs takes into account the amount of ventilated air and the difference in O_2_ and CO_2_ partial pressure between the air and the blood side in the alveoli. Gas transport in the peripheral tissues is illustrated only for the left leg for brevity. It takes into account the O_2_ consumption (*V*_*O2ll*_), the respiratory quotient (*RQ*), and the arterial and venous blood flow.

The cardiovascular model includes a representation of both atrial and ventricular chambers, the systemic, and the pulmonary circulation (see Figure 2), as described in detail before (Fresiello et al., 2016; Fresiello et al., 2017). In brief, atria and ventricles are modelled by a time-varying elastance model (Sagawa et al., 1988). During diastole, the ventricular compliance is represented by an exponential function while the atrial compliance by a linear function. Heart valves are represented with a diode and a resistance. The arterial and venous systemic circulation is a multicompartment system, with Windkessel models organized in series and in parallel to represent: the ascending and descending aorta, the upper body, the kidneys, the splanchnic circulation, lower limbs, superior, and inferior vena cava. Similarly, the pulmonary circulation is represented by two Windkessel models, for the arterial and the venous vessels, respectively.

#### 2.2.2 Baroreflex and metabolic peripheral control

The baroreflex model acts as a negative feedback control system taking pressure in the ascending aorta as input and controlling heart rate, cardiac contractility, peripheral vascular resistance, and venous tone as output. It reproduces the vagal and sympathetic activities based on two non-linear first order control systems (Ursino, 1998), further adapted to account for additional phenomena as baroreflex resetting occurring during exercise (Fresiello et al., 2016).

The metabolic peripheral control is also represented by a non-linear first order system (Fresiello et al., 2016), and acts locally on each vascular region of the systemic circulation where lack of oxygen is detected in the venous compartment. Both metabolic and sympathetic control act on the peripheral vessels and their combined effect is modelled according to the sympatholytic phenomenon, so that the sensitivity of a vascular region to the sympathetic activation reduces when a higher metabolic activity is detected, thus allowing for a more optimal blood flow regulation during physical exercise.

#### 2.2.3 Respiratory model

The respiratory model includes ventilation mechanics and control (Fresiello et al., 2016). The ventilation control adapts the ventilated air (tidal volume and frequency) to the O_2_ and CO_2_ partial pressures in the upper body. A model of ventilation mechanics accounts for the compliance of the lungs and the resistance of the airways and provides the pleural and intrathoracic pressure waveforms in relation to the ventilated air. The intrathoracic pressure is input into the cardiovascular system and affects the heart and the vessels within the thorax. The model of gas exchange in the lungs is represented by a mass balance equation that takes the volume of ventilated air and the pulmonary blood flow into account. Gas transport in the peripheral tissues is also described by a mass balance equation that considers the oxygen extraction rate, the respiratory quotient, and the arterial and venous blood flows in the considered vascular region (Fresiello et al., 2016).

### 2.3 *In vitro* hydraulic interface

**FIGURE 3:**
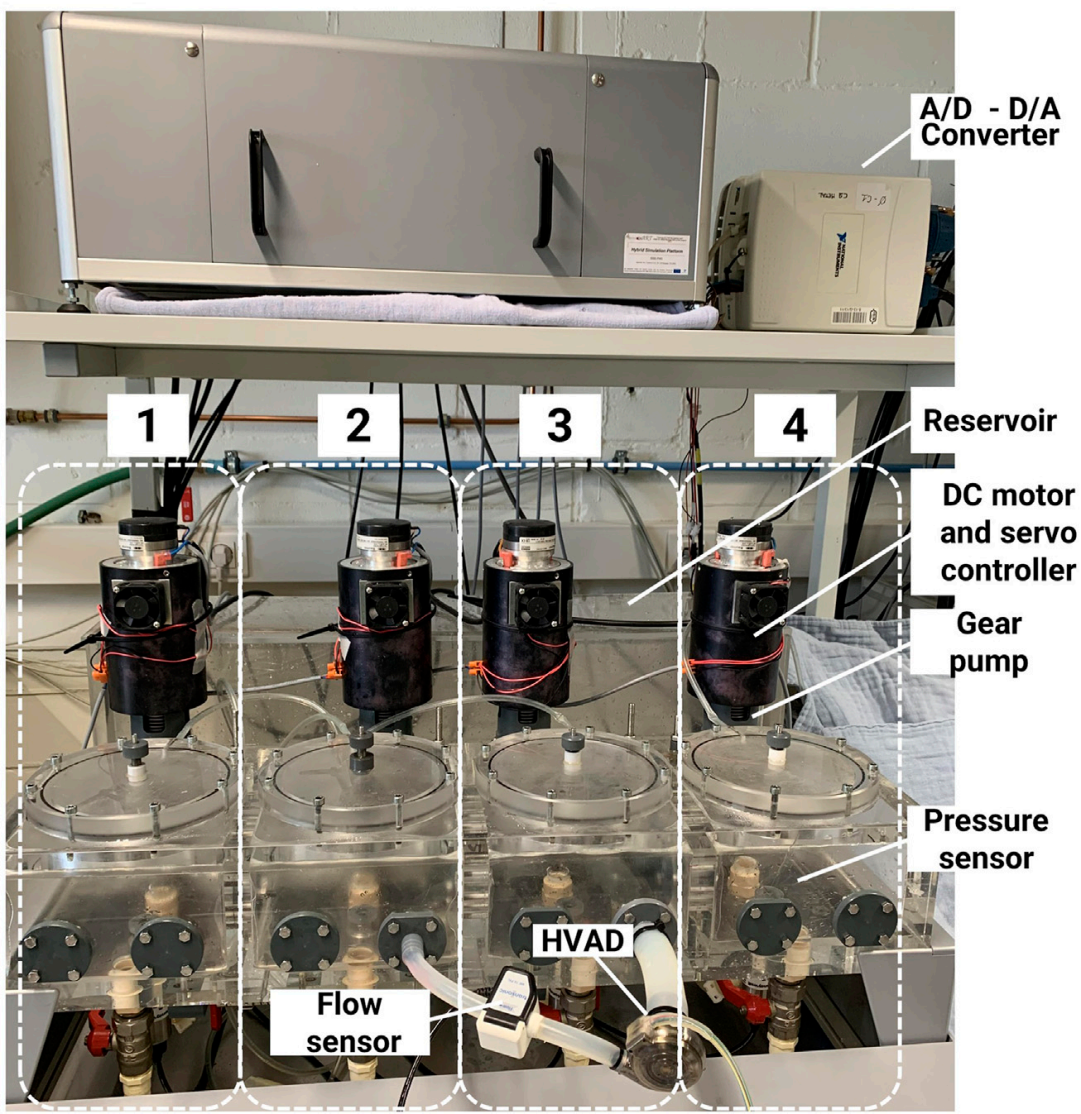
Hydraulic interface of the hybrid simulator. The system includes four hydraulic chambers, each actuated by a gear pump and a DC motor. The HVAD is connected between two chambers, one representing the left ventricle and the other the aorta.

The hydraulic interface was developed at the Nalecz Institute of Biocybernetics and Biomedical Engineering (Fresiello et al., 2014; Fresiello and Zieliński, 2020). The four hydraulic chambers are activated by DC motors and custom-made gear pumps that push liquid in/out each chamber from/to a reservoir in order to increase/decrease the pressure (Figure 3). A motor servo controller assures a linear relationship between the control voltage and the rotational speed of the motor. The gear pump, in turn, provides a flow proportional to the motor shaft angular velocity. The pressure in each chamber is monitored by a pressure sensor (PPG Honeywell, Columbus, OH, United States) and it is sent to the numerical model that in turn commands the flow to be produced by the gear pump. By this mechanism, the four chambers can mimic the pressure in up to four anatomical sites of the cardiovascular system, either cardiac or vascular ones. The number of chambers to be used and their configuration depends on the medical device to be tested (Fresiello and Zieliński, 2020). In this study, an LVAD is chosen as medical device in the case scenario, so two hydraulic chambers are configured as left ventricle and ascending aorta, respectively. The two hydraulic chambers exchange information with the *in silico* model in real-time: left ventricular pressure and flow, ascending aortic pressure and flow, and intrathoracic pressure. The connection between the *in vitro* interface and the *in silico* system is shown in Figure 1.

### 2.4 User interface

**FIGURE 4:**
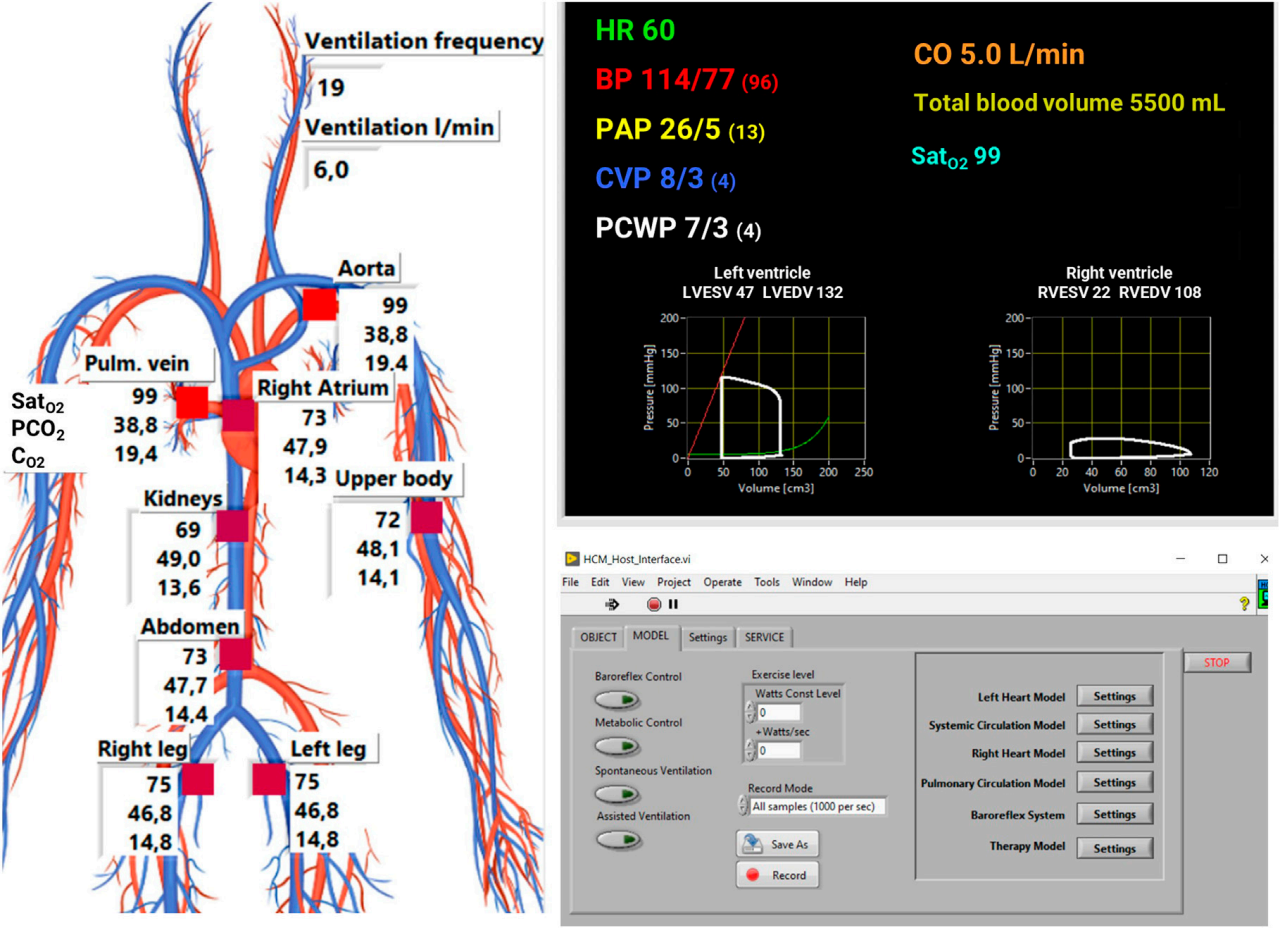
User interface of the simulator. Hemodynamic data are plot on the top right panel: heart rate (*HR*), systemic arterial pressure (*BP*), pulmonary arterial pressure (*PAP*), central venous pressure (*CVP*), pulmonary capillary wedge pressure (*PCWP*), cardiac output (*CO*), arterial oxygen saturation (*Sat_O2_*). Respiratory parameters (saturation of O_2_, partial pressure of CO_2_ and concentration of O_2_) are plot on the left panel. A dedicated window (bottom right panel) is used to activate control mechanisms, change cardiorespiratory parameters, and to input the exercise level either manually as a constant value or as a slope of increase over time.

The user interface is an application in LabVIEW™ that plots model output and sends parameters value to the cardiorespiratory model (Figure 4). It shows the main hemodynamic and respiratory variables of the simulated patients as pressures, volumes, O_2_, and CO_2_ content in different circulatory regions (see Figure 2). An additional window allows the user to change manually the cardiorespiratory lumped parameters and to insert different exercise levels to simulate. This window also enable to save data as waveforms with different sampling frequencies.

### 2.5 Clinical case study—left ventricular assist device

An implantable rotary blood pump (HVAD HeartWare, Medtronic Minnesota, MN, United States) was studied. The HVAD was connected to the hydraulic interface as shown in Figure 3 and a 10 PXL clamp-on flow sensor (Transonic, NY, United States) was attached to the outflow graft. The hydraulic chambers were filled with a mixture 35%–65% glycerol-water in weight at room temperature to match human blood viscosity (Segur and Oberstar, 1951). For patients with an LVAD, we considered a hemoglobin level of 13.2 g/dl (Fresiello et al., 2020), that corresponds to a whole blood viscosity of 3.39 cps (Ho, 2004).

#### 2.5.1 Clinical validation study

Data for model validation refers to two groups of clinical tests. Group1 refers to literature data on supine exercise tests at 44 W in LVAD patients where hemodynamic, blood, and pump parameters were collected at the beginning and at the end of physical exercise (Lai et al., 2020). Group2 refers to upright maximal exercise tests at 90 W in LVAD patients, where hemodynamic and respiratory parameters were recorded continuously throughout the entire exercise protocol.

##### 2.5.1.1 Group1—left ventricular assist device patients performing exercise test up to 44 W

The protocol of Group1 was already reported in Lai et al. (2020), briefly summarized: a total of 14 patients supported by an HVAD were collected at the St. Vincent’s Hospital, Sydney, Australia. Data included is covered by the study ethics approval HREC/13/SVH/32 and published in Lai et al. (2020). Mean arterial systemic pressure (*MAP*) was measured with an arterial Doppler-guided sphygmomanometer. Right atrial pressure (*RAP*), mean pulmonary arterial pressure (*PAPm*), and pulmonary capillary wedge pressure (*Pwedge*) were measured with a pulmonary artery catheter (CCOmbo; Edwards LifeSciences, Irvine, CA). Cardiac output (*CO*) was also monitored (Vigilance II Monitor; Edwards Lifesciences, Irvine CA). Mixed venous oxygen saturation (*Sat*_*vO2*_) was measured continuously (Vigilance II Monitor; Edwards LifeSciences). HVAD speed and estimated flow were collected from the device monitor. End-systolic and end-diastolic ventricular volumes (*LVESV*, *LVEDV*) were calculated using the Teichholz method from ventricular diameters measured with transthoracic echocardiography (Lang et al., 2015).

Data was collected in supine position at rest with the HVAD running at baseline speed 2,620 ± 89 rpm. Then patients performed an exercise test on a supine bicycle ergometer with incremental workload, from 0 to 44 ± 15 W. During the exercise test, the HVAD speed was progressively increased from baseline speed with steps of +80 rpm/2 min until a maximum 2,920 ± 90 rpm. Then, while exercising, HVAD speed was reduced to the baseline value.

##### 2.5.1.2 Group2—left ventricular assist device patients performing exercise test up to 90 W

The maximal exercise tests were performed at the Department of Cardiovascular Medicine of the University Hospitals Leuven. Data were collected retrospectively for all patients implanted between 2012–2022 with an HVAD, a HeartMate II or a HeartMate III (Abbott, Inc., IL, United States). The study was approved by the local Ethical Committee of the University Hospitals Leuven (S66789). Data refer to upright maximal exercise tests, performed by a cohort of 67 patients. Of these patients, 34 were implanted with a HM II, 21 were implanted with a HM III and 12 with an HVAD. Patients performed an exercise test on an ergometer bicycle (Ergometrics 800S, Ergometrics, Bitz, Germany). Patients started cycling at a workload of 10 W with an increase of 10 W/minute until exhaustion, defined as leg fatigue and/or dyspnea. Respiratory parameters as oxygen consumption (*V*_*O2*_), ventilation (*Ve*), and breathing frequency were acquired through a mask connected to a computerized system (Oxygen AlphaR, Jaeger, Mijnhardt, Bunnik, Netherlands). A 12-lead ECG was also acquired (Cardiosoft, CareFusion Coorporation, San Diego, California, United States) as well as non-invasive blood pressures through an arm cuff.

#### 2.5.2 Model parameters characterization: Generic left ventricular assist device patient

In this paragraph the characterization of the circulatory and respiratory simulator parameters is described. Model parameters were estimated using the data of Group1 at rest, expressed as mean values to obtain a “generic LVAD patient” profile.

Systemic and pulmonary vascular resistances (*SVR*, *PVR*) were calculated based on Ohm’s law and complying with clinical standards:

$$SVR=\frac{MAP-RAP}{CO}$$

$$PVR=\frac{PAPm-Pwedge}{CO}$$ (1)

Where *MAP* is the mean systemic arterial pressure, *RAP* is the mean right atrial pressure, *PAPm* is the mean pulmonary arterial pressure and *Pwedge* is the pulmonary capillary wedge pressure, *CO* is the cardiac output. The *SVR* was then repartitioned in the different circulatory districts reported in Figure 2 considering the distribution of total cardiac output: 23% for upper body, 22% for kidneys, 30% for splanchnic circulation, and 25% for legs (Heldt et al., 2002).

Ventricular volumes were used to characterize the end-systolic elastance (*Els*) and the filling characteristics of the left ventricle:

$$Els=\frac{MAP}{LVESV-{LV}_{0}}$$ (2)

where LV_0_ is the zero pressure filling volume considered to be equal to 0 mL to account for possible reverse remodeling of the ventricle due to the implantation of the HVAD (Fresiello et al., 2017). Right ventricular contractility parameter (*Ers*) is based on published data (Plehn et al., 2010).

The filling characteristics of the left ventricle was represented by an exponential relationship between ventricular pressure (*Plv*) and volume (*Vlv*):

$$Plv=al{\cdot \exp}^{bl\cdot Vlv}$$ (3)

Where *al* and *bl* are constant parameters estimated from *Plv* and *Vlv* at the end of diastole (*Pwedge* was considered as end-diastolic *Plv*). For patients of Group1 the LVEDV and *Pwedge* were measured repetitively at rest and during exercise. The filling characteristic was estimated by fitting these data with an exponential line. Then, an average exponential line among all patients was calculated with relative *al* and *bl* parameters.

Parameters of the respiratory systems (airway resistance and elastance of the lungs), baroreflex control, metabolic control and respiratory control were set as reported before (Fresiello et al., 2016).

#### 2.5.3 Model parameters characterization: Exercise simulation

To simulate cycling exercise, the simulator takes the increase in oxygen consumption and respiratory quotient as an input parameter that accounts for the increased metabolic activity in the legs.

For the Group1 exercise test at 44 W a *V*_*O2*_ of 717 mL/min was considered. This was not measured directly but estimated from patients data of Group1 according to the Fick principle:

$$V_{O2}=CO\cdot \left(C_{aO2}-C_{vO2}\right)$$ (4)

where C_aO2_ and C_vO2_ are the arterial and venous oxygen concentrations calculated as it follows:

$$C_{O2}=Hb\cdot 1.36\cdot \frac{{Sat}_{O2}}{100}+0.0031\cdot PO_{2}$$ (5)

where Hb is the hemoglobin concentration, *Sat*_*O2*_ and *PO*_*2*_ are the saturation and the partial pressure of oxygen in the blood, respectively. As venous oxygen saturation the average value measured on patients was considered. For the arterial oxygen saturation, blood was assumed to be fully oxygenated when exiting the pulmonary circulation.

**FIGURE 5:**
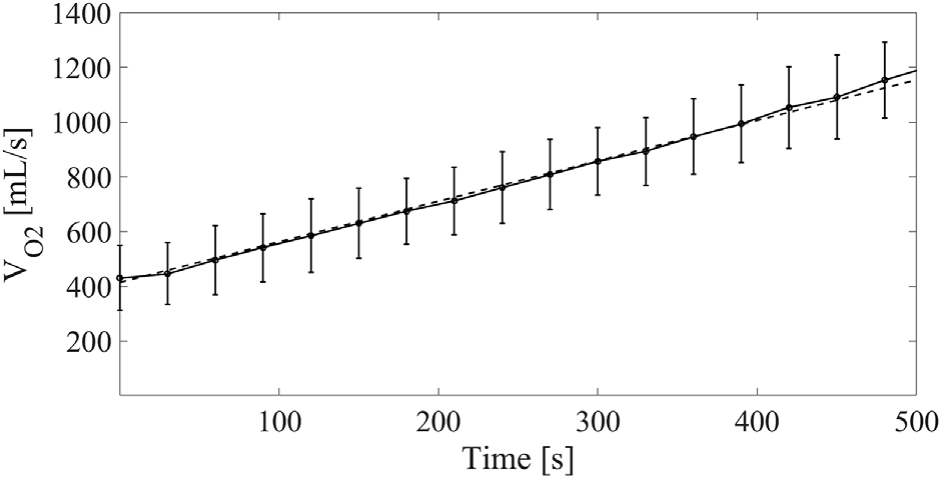
Continuous line: oxygen consumption measured on LVAD patients at different time points for the Group2 exercise test from 10 to 90 W. Dashed line: linear model of V_O2_ increase fed into the cardiorespiratory simulator.

For Group2, the time evolution of *V*_*O2*_ from 10 to 90 W exercise was measured continuously. Using these data, an average trendline of *V*_*O2*_ increase over time was considered as reported in Figure 5.

### 2.6 Simulation procedure

**FIGURE 6:**
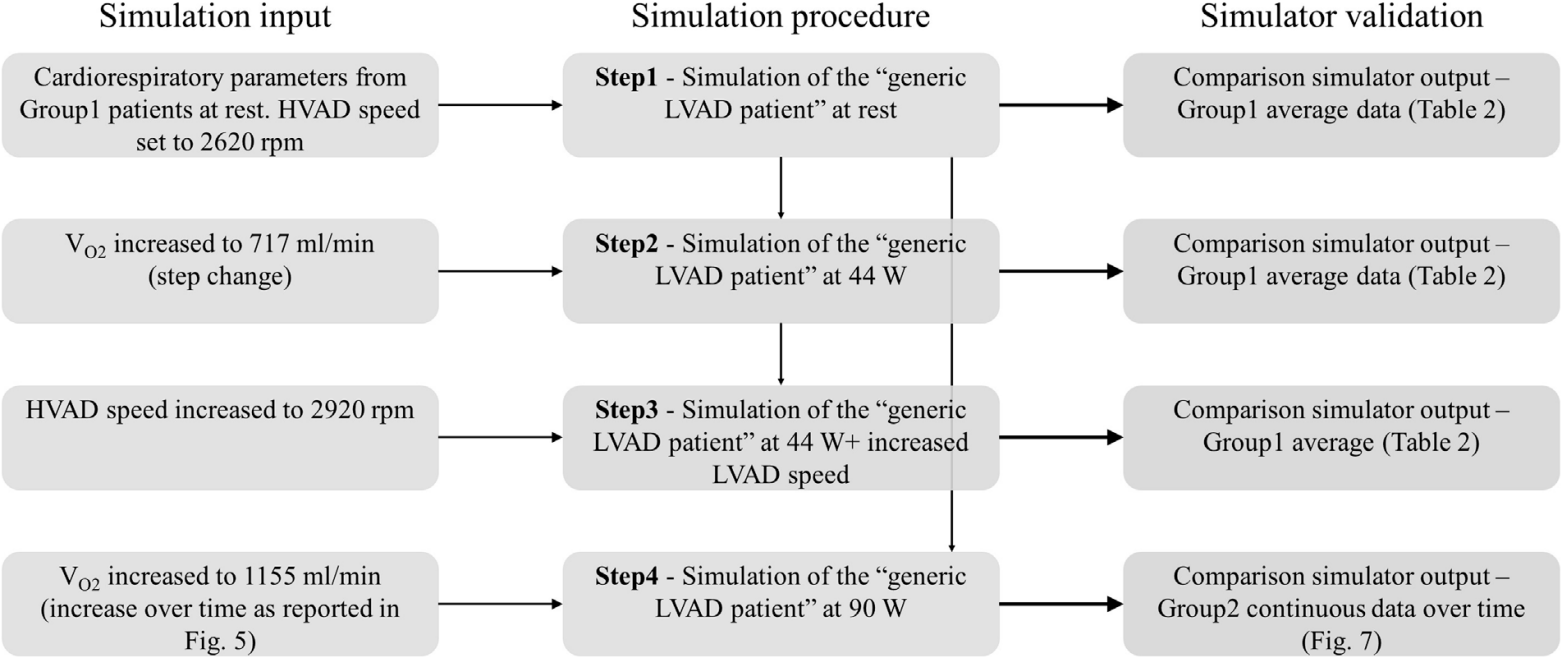
Workflow of the simulation steps for the Group1 and Group2 exercise tests.

Simulations consisted of 4 consecutive steps, summarized in Figure 6. They are described below in details:

Step 1 —Simulation of rest condition.

The parameters of the cardiorespiratory simulator were manually tuned to reproduce the generic LVAD patient at rest. The HVAD device was connected to the simulator and the speed was set to 2,620 rpm, same as in patients. Once the cardiorespiratory system reached steady state, data were recorded.

Step 2 —Simulation of Group1 exercise at 44 W.

From Step 1, exercise at 44 W was simulated by increasing *V*_*O2*_ to 717 mL/min. When steady state condition was reached, data were recorded.

Step 3 —Simulation of Group1 exercise at 44 W with increased HVAD speed.

From Step 2, HVAD speed was increased to 2,920 rpm. When steady state condition was reached, data were recorded.

Step 4 —Simulation of Group2 exercise at 90 W.

Starting from the rest condition of Step 1, the evolution from rest to 90 W exercise was simulated by increasing *V*_*O2*_ over time as reported in Figure 5. Data were collected during the entire experiment.

It is important to underline that cardiorespiratory parameters were manually tuned only at Step 1, to reproduce the generic LVAD patient at rest. For Step 2-3-4, the cardiorespiratory system automatically evolved from rest to exercise condition as a reaction to the increased *V*_*O2*_, thanks to the physiological control mechanisms implemented.

### 2.7 Data post-processing

Clinical data at Step 1-2-3 were expressed as mean and standard deviation over the patient population at rest and at exercise. Similarly, simulation data at Step 1-2-3 were analyzed and mean and standard deviations values were calculated over 15 simulated heart beats to average the effect of ventilation on cardiac variables. The difference between simulated data and clinical measurements was calculated in terms of percentage error. The authors considered 10% and 20% as thresholds for good agreement between simulations and clinical data, for hemodynamic and echocardiographic data, respectively. These thresholds were decided considering the error usually affecting these clinical measurements (Jenkins et al., 2004; Chase et al., 2010). Concerning the HVAD flow, it is difficult to set an error threshold since the flow is not directly measured in the clinics but estimated from pump speed and power consumption, The estimation depends on patient’s blood viscosity level that is not always updated into the controller.

Clinical data at Step 4 acquired during the period of increasing workload from 10 W till 90 W were isolated and interpolated as to achieve equal sampling intervals. This data was then analyzed in terms of mean and standard deviations over the patient cohort for each considered sampling time. Similarly, the trends of cardiovascular variables over time obtained from simulations were plot. The difference between simulated and clinical waveforms was calculated in terms of root mean square error.

## 3 Results

The parameters of the cardiorespiratory simulator are reported in Table 1. Parameters at rest condition were manually input into the simulator. Parameters at exercise are the result of the physiological control mechanisms implemented in the cardiorespiratory simulator: *HR*, *Els*, *Ers,* and venous tone change under the baroreflex control, *SVR* changes under the baroreflex and metabolic controls, ventilation is regulated according to the arterial *PO*_*2*_ and carbon dioxide partial pressure (*PCO*_*2*_) sensed in the upper body. Parameters as *PVR*, *al*, *bl*, *ar*, *br* remain constant from rest to exercise since no control mechanism of the computational model acts on them.

**TABLE 1:** List of parameters of the cardiorespiratory simulator: V_O2_, oxygen consumption; *PVR*, pulmonary vascular resistance; *SVR*, systemic vascular resistance; *al* and *bl* (*ar* and *br*), filling characteristic of the left (right) ventricle; *Els* (*Ers*), end-systolic elastance of the left (right) ventricle; HR, heart rate; *Ve* ventilation.

	V_O2_	HVAD speed	PVR	SVR	al	bl	ar	br	Els	Ers	HR	Ve	Frequency Ve	Tidal volume
	mL/min	rpm	Wood units	Wood units	mmHg	mL^−1^	mmHg	mL^−1^	mmHg/mL	mmHg/mL	bpm	L/min	n/min	mL
Step 1—rest	332	2,620		13.9					0.58	0.61	79	10.6	21	512
Step 2—Group1 exe. (44 W)	717	2,620		13.2					0.63	0.66	98	22.5	25	900
Step 3—Group1. exe. (44 W) + increased HVAD speed	717	2,920	1.67	13.2	1.000	0.017	0.044	0.045	0.62	0.66	97	25.0	25	1,000
Step 4—Group2. exe. (90 W) (peak values)	1,155	2,620		10.1					0.67	0.7	119	38.5	28	1,360

### 3.1 Comparison simulation—Group1 clinical data

In Table 2, the output of the simulator for Step 1-2-3 is compared to the clinical data of Group1 in terms of hemodynamic values, *Sat*_*vO2*_ and LVAD parameters. The simulation data are in agreement with the clinical data meaning that the simulator can capture the general hemodynamic and respiratory status of LVAD patients at both rest and exercise conditions. For the pulmonary pressure and wedge pressure, the simulator underestimates their increase observed on patients at exercise. HVAD flow measured on the simulator is also in line with clinical data, indicating that the device overall interacts with the simulated heart and aorta in a similar manner compared to patients.

**TABLE 2:** Comparison between simulated data and clinical data of the 14 HVAD patients of Group1: *MAP*, mean arterial pressure; *RAP*, right atrial pressure; *PAPm*, mean pulmonary arterial pressure; *Pwedge*, pulmonary capillary wedge pressure; *CO*, cardiac output; *Sat_vO2_*, mixed venous oxygen saturation; *LVESV*, end-diastolic left ventricular volume.

		HVAD flow	MAP	RAP	PAPm	Pwedge	CO	Sat_vO2_	LVEDV
		L/min	mmHg	mmHg	mmHg	mmHg	L/min	%	mL
	Clinical data	4.6 ± 0.8	84 ± 8	9 ± 4	23 ± 6	15 ± 5	4.9 ± 1.1	63 ± 5	173 ± 77
Step 1—rest	Simulated data	4.8 ± 0.0	88 ± 0	11 ± 1	26 ± 1	16 ± 1	4.8 ± 0.1	58 ± 0	181 ± 1
	%Error	5.3	5.1	23.2	12.7	6.7	−1.3	−8.3	4.6
	Clinical data	5.9 ± 0.9	94 ± 9	17 ± 5	38 ± 8	30 ± 8	6.3 ± 1.5	27 ± 8	173 ± 77
Step 2—Group1 exe. (44 W)	Simulated data	5.0 ± 0.0	93 ± 1	13 ± 2	31 ± 1	20 ± 2	6.0 ± 0.2	27 ± 0	190 ± 1
	%Error	−15.3	−1.1	−23.2	−18.0	−32.7	−4.1	0.2	9.8
	Clinical data	6.7 ± 1.0	94 ± 8	18 ± 6	37 ± 8	27 ± 8	5.7 ± 1.1	27 ± 10	173 ± 77
Step 3—Group1 exe. (44 W) + increased HVAD speed	Simulated data	6.1 ± 0.0	95 ± 1	13 ± 2	30 ± 1	18 ± 2	6.2 ± 0.1	28 ± 0	182 ± 1
	%Error	−8.9	1.5	−26.8	−18.1	−33.2	9.1	5.0	5.2

### 3.2 Comparison simulation—Group2 clinical data

**FIGURE 7:**
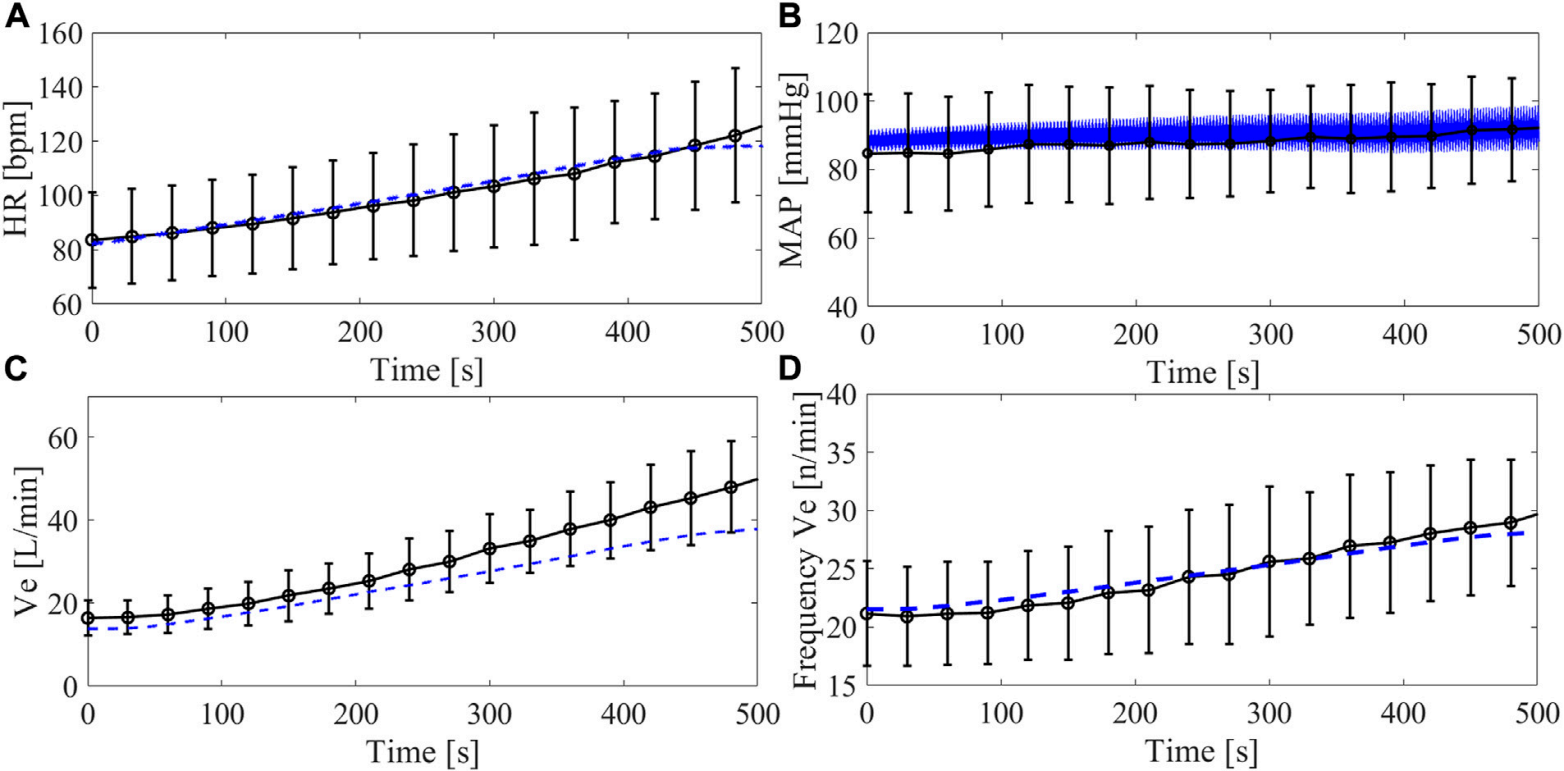
Trend of cardiorespiratory parameters over time during the Group2 exercise test (from 10 to 90 W): heart rate **(A)**, arterial systemic pressure **(B)**, ventilation **(C)**, ventilation frequency **(D)**. Black lines refer to clinical data from Group2 (average and standard deviation), blue lines refer to simulations data. For the arterial systemic pressure, average values are shown for the clinical data and waveforms are reported for the simulations.

The comparison between the simulation and the clinical data of the Group2 exercise tests (Step 4) is reported in Figure 7. Data refer to the trends of *HR*, systemic arterial pressure, *Ve* and breathing frequency over time during the entire simulation from rest to peak exercise. The calculated root mean square error between clinical and simulated data was 2 bpm for the *HR*, 3.0 mmHg for the *MAP*, 5.4 L/min for *Ve*, and 0.7 n/minute for the breathing frequency. Overall, simulations are in agreement with clinical data, indicating that the simulator can reproduce the dynamic changes of the cardiorespiratory parameters from rest to exercise. For *Ve*, simulation and clinical data differ for higher values of *V*_*O2*_. Concerning the systemic arterial pressure, the *MAP* is reported for the clinical data while for the simulation the waveform is plot. It is evident that the simulated systemic arterial pressure increases in pulsatility with exercise level, a sign that the left ventricle starts to eject.

### 3.3 Left ventricle—left ventricular assist device interaction

**FIGURE 8:**
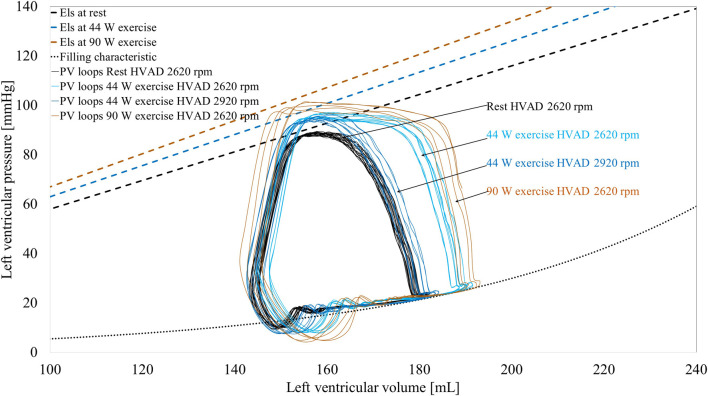
Simulated ventricular pressure-volume loops of the left ventricle supported by an HVAD for different conditions. Also the end-systolic elastance (*Els*) and the diastolic filling curves of the left ventricle are shown.

**FIGURE 9:**
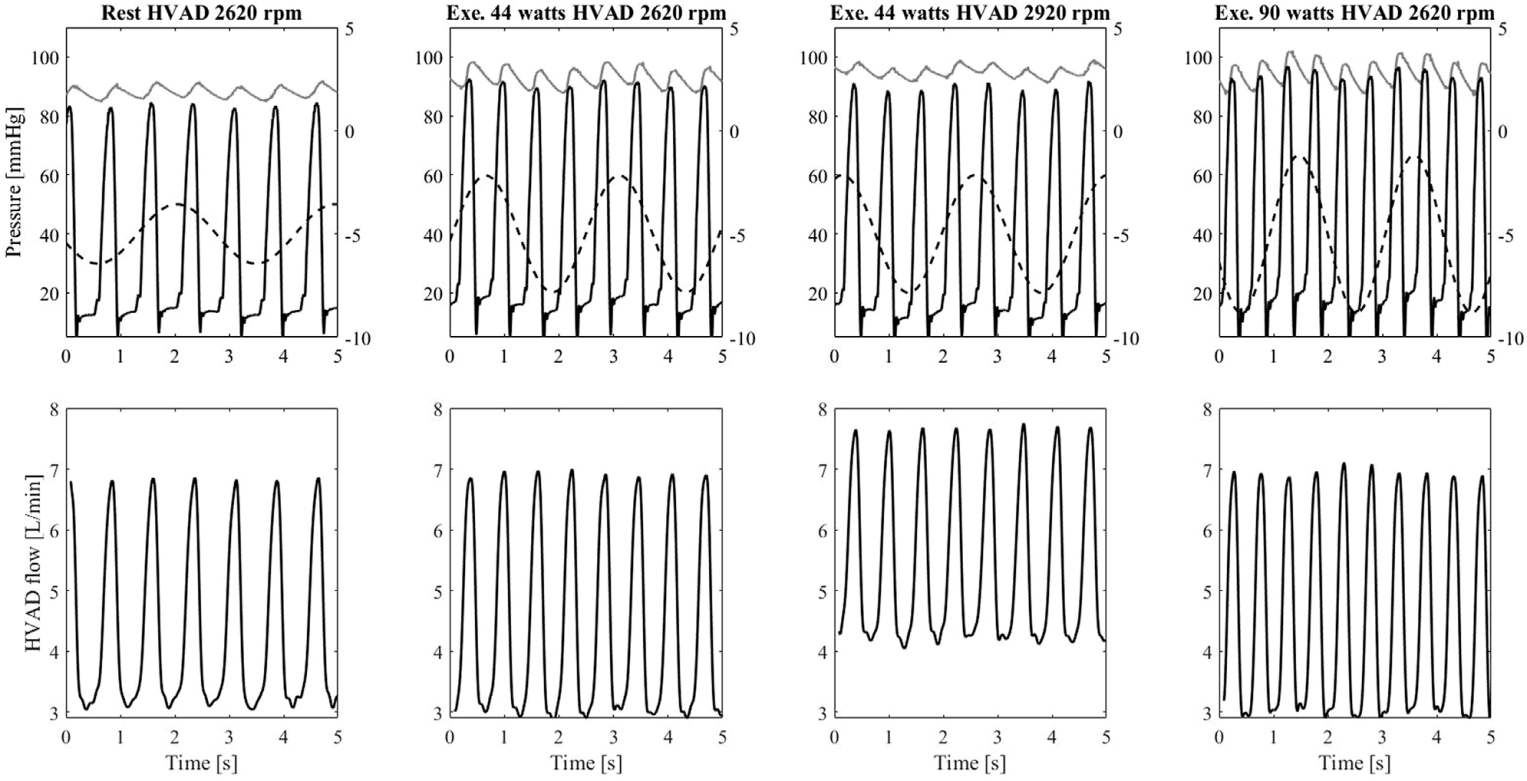
Upper panel: left ventricular pressure (black), ascending aorta pressure (grey), and intrathoracic pressure (dashed line plot against the secondary *y*-axis) measured on the simulator. Lower panel: flow waveforms measured from the HVAD connected to the simulator. Data from the left to the right column refers to: rest condition with HVAD at baseline speed Group1 exercise (44 W) with HVAD at baseline speed, Group1 exercise (44 W) with HVAD at increased speed, Group2 exercise (90 W) with HVAD at baseline speed.

The interaction between the left ventricle and the LVAD is represented as pressure – volume loops in Figure 8. The simulator can reproduce hydraulically the different diastolic and systolic mechanical properties of the left ventricle as well as the changes in preload and afterload due to the LVAD and exercise. During exercise, the venous return increases, provoking a rightward shift of the pressure-volume loops. Also, the Els increases due to the positive inotropic effect exerted by the baroreflex control mechanism. As a result, the left ventricle develops higher pressure in systole. By increasing HVAD speed to 2,920 rpm, the ventricle is more unloaded and the original pressure-volume loop is partially restored and shifted towards the left side of the panel. These changes of ventricular condition and LVAD speed, in turn affect the aortic valve opening. To better clarify this phenomenon, in Figure 9 the waveforms of left ventricular and aortic pressure are reported. At rest the aortic valve is closed while during exercise the aortic valve starts to open. By increasing LVAD speed during exercise, a full support condition is restored.

Figure 9 shows also the effect of the intrathoracic pressure that directly affects the ventricular and aortic pressures. This in turn affects the HVAD flow that shows a profile modulated by the ventilation. During exercise, both tidal volume and ventilation frequency increase. The intrathoracic pressure changes are amplified leading to more pronounced effects on the HVAD flow profile.

## 4 Discussion

### 4.1 The need for high-fidelity simulators in mechanical circulatory support testing

This work is a first step towards a more reliable, comprehensive and clinically validated cardiorespiratory test bench for the assessment of the hemodynamic performance of MCSs within a realistically simulated human body. Such an assessment can be very demanding when testing not only the MCS itself, but also monitoring and control features embedded in the technology that critically interact with a patient-specific pathophysiological condition. It is indeed a desirable milestone in healthcare to bring more “intelligent” medical devices to the clinics, able to adequately detect the patient’s status and adapt the level of support accordingly. The MCSs in particular should provide the proper level of support to restore hemodynamics as well as ensure adequate oxygenation to peripheral organs and tissues. In this regard, several algorithms are under development for different MCS technologies as LVADs, percutaneous temporary circulatory support devices and total artificial hearts (Naito et al., 2017; Chia-Wei Sun, 2020; Maw et al., 2020). It is very difficult to test these complex physiological control mechanisms in detail in a clinical setting, due to obvious safety and ethical issues. *In silico* and *in vitro* simulators offer a versatile platform for optimization of design and testing of these algorithms, by reproducing a large variety of clinically relevant scenarios that challenge device operation and control (Petrou et al., 2019, 2018; Schmid Daners and Dual, 2020). In this paper we tested a stepwise increase of HVAD speed, operated manually. The intent was to reproduce exactly what was performed in clinical practice in order to prove that the simulator can reliably capture the effects of LVAD speed change that impact on the human cardiovascular system. This approach preludes the testing of more sophisticated control algorithms in the future, enabling to adapt LVAD speed dynamically and proportionally to patient-specific physiological needs.

### 4.2 Testing the interaction of the mechanical circulatory support with the native heart

An important aspect to carefully evaluate in MCSs is its interaction with the supported cardiovascular site, particularly the left ventricle in case of an LVAD. This implies that the simulator should accurately reproduce the functional properties of the failing ventricle. Moreover, the simulator should feature adaptability to these ventricular properties, since they can very dynamically change over time: inotropic influences can be mimicked instantaneously via the autonomic controls, while cardiac recovery or (reverse) remodeling processes that may occur over time while on LVAD support should also realistically be accounted for (Madigan et al., 2001; Phan et al., 2016).

The simulator we propose also offers a large adaptability to reproduce diastolic and systolic ventricular properties, including non-linear features and thus enables to create personalized parameters with values in the range that is clinically observed in LVAD patients. The autonomic control regulates the ventricular contractility, as it is shown in Table 1 with a positive inotropic effect on both left and right ventricles during exercise (*Els* 0.58-0.63-0.62-0.67 mmHg/mL and *Ers* 0.61-0.66-0.66-0.7 mmHg/mL). Also, the positive chronotropic effect is reproduced, so heart rate increases from 79 bpm at rest to 98–119 bpm at different levels of exercise.

The simulated changes in ventricular properties reveal the well-appreciated chronotropic and inotropic impairment of the left ventricle, as a result of a chronic and severe heart failure condition that persists after the implantation of an LVAD (Loyaga-Rendon et al., 2015). The chronotropic reserve predicted by the simulator is 40 bpm, in line with 37 ± 22 bpm reported by Moss et al. (2020). Likewise, the inotropic reserve is limited and not sufficient to counteract the increase in wedge pressure (see Table 2), an inherent characteristic in this specific patient population noticed in different clinical studies on LVAD patients (Muthiah et al., 2015; Moss et al., 2020). The simulator underestimates the increase in wedge pressure and pulmonary arterial pressure from rest to exercise compared to what observed in the clinics (Table 2). A possible reason of this discrepancy is the relatively simplistic model used in the simulator for the pulmonary circulation, that does not account for phenomena as pulmonary congestion and its effect on lungs resistance and compliance.

In order to provide clinically meaningful data, it is important that a simulator realistically reproduces the response of the myocardium to pathophysiological stimuli and to account for impairments of responses as a result of the underlying cardiovascular diseases. Capture these phenomena is of pivotal importance when aiming to design and test an MCS able to compensate for the non-physiological cardiac responses to changes in the body condition. In this context, several clinical studies reported a limited support of the LVAD during maximal exercise: a rather modest LVAD flow increase (<1.5 L/min on average), insufficient with respect to the body’s demands (Muthiah et al., 2015; Gross et al., 2019; Moss et al., 2020). In general, the left ventricle resorts in contributing itself to cardiac output (within its limits), something observable from the increase in the occurrence of aortic valve opening when switching from rest to exercise (Brassard et al., 2011; Gross et al., 2019). Intriguingly, our simulator is capable of predicting this phenomenon correctly under LVAD support: the left ventricle, not ejecting at rest, starts to eject during exercise thus contributing to accommodate a higher cardiac output (Figure 9). This ultimately translates into a higher blood pressure pulsatility in the systemic circulation as shown in Figure 7B.

### 4.3 Testing the interaction of the mechanical circulatory support with peripheral tissues

When testing an MCS, a simulator should also reproduce the changes in the circulatory system that ultimately translate in perfusion needs in different circulatory regions. Indeed, the representation of body oxygenation is a key element when supporting the design of a physiological MCS control that would properly unload the heart as well as perfuse the whole body. Thus, a simulator should ideally include the whole circulatory system and feature its intricate regulatory mechanisms. In our simulator, the autonomic and the metabolic peripheral control mechanisms are implemented, together with the sympatholytic effects that allows to capture the response of exercising and non-exercising vascular compartments to physical exertion, individually. As an outcome, the total SVR progressively decreases from Step 1 (13.9 Wood units) to Step 2-Step 3 (13.2 Wood units) to Step 4 (10.1 Wood units). This SVR drop, reported in Table l is mainly the result of vasodilation in the exercising vascular compartments (legs for the proposed exercise test), where a low oxygen content triggers the sympatholytic effect.

The simulator was able to predict *Sat_vO2_* with a good level of agreement, as reported in Table 2 (from 58% at Step 1, to 27% at Step 2 to 28% at Step 3). It is likely to assume that the changes in *Sat*_*vO2*_ reflect the changes in legs venous oxygen saturation mostly (Sullivan et al., 1989). So, we can infer that the simulator is capable to capture the level of oxygenation, perfusion and vasodilation at a systemic level (Table 2) as well at the exercising vascular regions locally.

### 4.4 Testing the interaction of the mechanical circulatory support with the respiratory system

The implementation of the respiratory model and its full integration with the cardiovascular system is particularly challenging to be implemented *in vitro*. Previous mock loops have included the effect of ventilation on the cardiovascular system by either changing the *PVR* value cyclically (Love et al., 2014), or by imposing an intrathoracic pressure with a programmable proportional pneumatic valve (Vukicevic et al., 2013). In both these simulators, ventilation frequency and tidal volumes were manually set. In our simulator, the ventilation acts on the heart and vessels inside the thorax and is automatically regulated by the dedicated control according to the arterial PO_2_ and PCO_2_ in the upper body. As such, ventilation increases from Step 1-2-3-4 both in terms of frequency (21-25-25-28 n/minutes) and tidal volume (512-900-1,000–1,360 mL). This increase is in line with that measured in the clinics (Figure 7) except at the latter half of the maximal exercise test, when the simulated ventilation is lower than the one measured on patients. This discrepancy is probably due to the switch from aerobic to anaerobic metabolism, expected to occur at about 40% of the maximal *V*_*O2*_ in LVAD patients (Fresiello et al., 2020). The simulator does not account for anaerobic metabolism and lactic acid production and the consequent disproportionate increase in ventilation (Albouaini et al., 2007)*.*

These changes in ventilation affect the intrathoracic pressures, and in turn the transmural pressures across the vessels within the thorax (Figure 2) and finally the LVAD flow. Figure 9 gives an example of the modulation effect that ventilation has on the LVAD flow profile. The ability to capture these features is important when testing monitoring and control algorithms for MCS, that aim at identifying parameters useful to infer a patient-specific condition. Many attempts are ongoing on extrapolating relevant pathophysiological features of individual patients starting from MCS signals (e.g., current, flow) (Moscato et al., 2021). A fully closed-loop cardiorespiratory simulator allows to represent heart-lung interaction and adds more realism concerning the variability to be expected from the MCS signals. In this regard, the simulator can help testing the robustness of monitoring algorithms and defining tolerance limits beyond which a change in signal has to be labelled as non-physiological and potentially detrimental for the patient.

### 4.5 Testing dynamic aspect of mechanical circulatory support performance

To operate as a comprehensive and versatile test bench for MCS controls, a simulator should also capture the dynamicity of changes of the cardiovascular system from status A to status B. This is important when testing control algorithms aimed at adapting the MCS performance to the patient’s needs. Figure 7 shows the dynamic evolution of our proposed cardiorespiratory simulator during maximal exercise. In mock loops published so far, exercise is usually simulated with stepwise changes of cardiovascular parameters operated manually and that do not reproduce the adaptation of body to exercise dynamically (Timms et al., 2011). Petrou et al. (2019) proposed a sophisticated hybrid simulator with a closed-loop exercise model able to reproduce the dynamic evolution of the cardiovascular system from rest to exercise. The model includes autonomic controls, but no respiratory model, so the metabolic vasodilation is a function of Metabolic Equivalent of Tasks and not of the real perfusion status of the circulatory regions.

## 5 Conclusion

We developed a closed-loop cardiorespiratory simulator in a real-time mixed *in silico*—*in vitro* environment. The *in silico* component retains most of the physiological model, the *in vitro* component acts as a hydraulic interface to connect a cardiovascular medical device as the ventricular assist device considered in this study and test its performance with the simulated human body. The simulator can reproduce complex scenarios as severe heart failure and is physiologically controlled, so it can recreate the dynamic adaptation of the human body over time and between different conditions, e.g., adaptation from rest to exercise. Validation against clinical data show that the obtained simulations are realistic in terms of cardiovascular and respiratory parameters, as well as in terms of interaction with the considered medical device. Overall, this work is a step forward towards the realization of a comprehensive and versatile test bench for cardiovascular medical devices and their monitoring and control algorithms.

## 6 Study limitations

The cardiorespiratory does not include coronary circulation and pulmonary hypoxic vasoconstriction. These features will be added in the future to account for additional cardiorespiratory diseases. The model does not account for anaerobic metabolism, a feature that will be included in the future to better reproduce prolonged physical exertion.

Clinical data of maximal exercise tests refer to patients supported by different LVAD types (HVAD, HM II, and HM III). For simplicity, simulations were run with the HVAD only, assuming the 3 LVADs would elicit similar hemodynamic outcome. This is in line with previous studies from our group where no differences were reported in terms of exercise capacity among patients supported by the 3 LVADs (Fresiello et al., 2020).

It goes without saying that a simulator able to serve the purpose of MCS testing, should offer a broader set of pathophysiological conditions than what presented in this work. We chose to present rest and exercise conditions as exemplary cases to prove the adaptability and versatile nature of the simulator in covering very different hemodynamic and respiratory statuses, and in capturing the main physiological mechanisms responsible for the transition from one status to another. Further efforts will be conducted to extend the range of validation of the simulator towards additional case scenarios and pathologies.

## Data Availability

The original contributions presented in the study are included in the article, further inquiries can be directed to the corresponding author.

## Abbreviations

*al/ar*, filling characteristic constant of the left/right venticle; *bl/br*, filling characteristic constant of the left/right venticle; *C*_*aO2*_, arterial oxygen concentration; *C*_*vO2*_, venous oxygen concentration; *CO*, total cardiac output; *Els/Ers*, left/right ventricular end-systolic elastance; *Hb*, hemoglobin concentration; *HR*, heart rate; *LVAD*, left ventricular assist device; *LVEDV*, end diastolic left ventricular volume; *LVESV*, end systolic left ventricular volume; *LV*_*0*_, zero pressure filling volume; *MAP*, mean arterial systemic pressure; *PAPm*, mean pulmonary arterial pressure; *Plv*, left ventricular pressure; *PVR*, pulmonary vascular resistance; *Pwedge*, pulmonary capillary wedge pressure; *PO*_*2*_, partial pressure of oxygen; *RAP*, mean right atrial pressure; *Sat*_*O2*_, oxygen saturation; *Sat*_*vO2*_, mixed venous oxygen saturation; *SVR*, systemic vascular resistance; *Ve*, ventilation; *V*_*O2*_, oxygen consumption.
